# Interpreting the results of patient reported outcome measures in clinical trials: The clinician's perspective

**DOI:** 10.1186/1477-7525-4-62

**Published:** 2006-09-14

**Authors:** Holger J Schünemann, Elie A Akl, Gordon H Guyatt

**Affiliations:** 1 Division of Clinical Research Development and INFORMAtion Translation, Department of Epidemiology, Istituto Regina Elena/Italian National Cancer Institute Rome, Rome, Italy; 2 Department of Medicine, University at Buffalo, New York, Buffalo, USA; 3 Department of Clinical Epidemiology and Biostatistics, McMaster University; Hamilton, Ontario, Canada; 4 Department of Medicine, McMaster University; Hamilton, Ontario, Canada

## Abstract

This article deals with the problem of interpreting health-related quality of life (HRQL) outcomes in clinical trials. First, we will briefly describe how dichotomization and item response theory can facilitate interpretation. Based on examples from the medical literature for the interpretation of HRQL scores we will show that dichotomies may help clinicians understand information provided by HRQL instruments in RCTs. They can choose thresholds to calculate proportions of patients benefiting based on absolute scores or change scores. For example, clinicians interpreting clinical trial results could consider the difference in the proportion of patients who achieve a mean score of 50 before and after an intervention on a scale from 1 to 100. For the change score approach, they could consider the proportion of patients who have changed by a score of 5 or more. Finally, they can calculate the proportion of patients benefiting and transform these numbers into a number needed to treat or natural frequencies. Second, we will describe in more detail an approach to the interpretation of HRQL scores based on the minimal important difference (MID) and proportions. The MID is the smallest difference in score in the outcome of interest that informed patients or informed proxies perceive as important, either beneficial or harmful, and that would lead the patient or clinician to consider a change in the management. Any change in management will depend on the downsides, including cost and inconvenience, associated with the intervention. Investigators can help with the interpretation of HRQL scores by determining the MID of an HRQL instrument and provide mean differences in relation to the MID. For instance, for an MID of 0.5 on a seven point scale investigators could provide the mean change on the instrument as well as the proportion of patients with scores greater than the MID. Thus, there are several steps investigators can take to facilitate this process to help bringing HRQL information closer to the bedside.

## Background

This article deals with the problem of interpreting health-related quality of life (HRQL) outcomes from clinical trials. The alternative titles that we had in mind for this paper exemplify the problem: "The great merit of simple-minded dichotomies for clinicians" or even more provocative, "The great merit of dichotomies for simple-minded clinicians." These possible titles reflect the fact that clinicians have difficulties understanding quality-of-life measures because of the numerous available instruments and their diversity in items, response options, lack of familiar units, and approaches to aggregation. We propose that dichotomies can help with understanding HRQL instruments.

In this article we will offer some possible strategies based on examples. First, we will briefly describe how dichotomization and Rasch analysis, a particular form of analysis according to item response theory (IRT), can facilitate interpretation [1,2]. We will describe how researchers can improve the presentation of HRQL outcome measures and how clinicians can use intuitive thresholds to interpret HRQL outcomes. Clinicians can use thresholds that either refer to an absolute score (e.g. clinicians can consider patients above a certain score as having achieved the outcome) or a change in score (e.g. clinicians can consider patients' HRQL as having improved or deteriorated if they achieve a certain change in score on an instrument of interest). For the absolute score, imagine a trial using the Short Form-36 (SF-36) HRQL instrument as the primary outcome. Clinicians interpreting clinical trial results could consider the proportion of patients who achieve a mean score of 50 before and after an intervention. For the change score approach, they could consider the proportion patients who have changed by a score of 5 or more. In the following text, we will describe these approaches and provide additional examples from the medical literature. While we will provide examples for the use of item response theory, we will not discuss details of the statistical methods. Interested readers should consult specialized texts for an in-depth understanding.

Second, we will suggest an approach to the interpretation of HRQL scores based on the minimal important difference (MID) [3-6]. The MID is the smallest difference in score in the outcome of interest that informed patients or informed proxies perceive as important, either beneficial or harmful, and that would lead the patient or clinician to consider a change in the management [3,6]. We place a greater weight on the preferences of informed patients than clinicians in studying the MID [6,7]. To further qualify this definition of the MID, only if informed patients cannot make decisions about the management of their disease, or if patients prefer informed proxies to make these decisions, would one consider the MID estimates of informed proxies. In addition, any change in management will depend on the downsides, including cost and inconvenience, associated with the intervention.

### How Do Investigators Present Health-Related Quality-of-Life Information Currently?

To highlight the problem, we describe an example of the failure to present HRQL in a transparent and understandable way to clinicians. In a trial including 553 patients with psoriasis, a group of investigators evaluated the impact on HRQL of alefacept, a fusion protein that inhibits T-cell activation and promotes apoptosis of CD2+ T cells that play a role in psoriasis, on HRQL [8]. They used the Dermatology Life Quality Index (DLQI), Dermatology Quality of Life Scales (DQOLS), and SF-36. The authors describe that alefacept significantly reduced (improved) mean DLQI scores compared with the placebo. The improvements were 4.4 points vs. 1.8 at 2 weeks just after the last dose (P < 0.0001) and 3.4 vs. 1.4 at 12 weeks after the last dose (P < 0.001). They further mention that a group of patients who received two courses of alefacept experienced additional enhancement of quality-of-life measures during the second course.

In the authors' opinion, the data from the SF-36 survey confirmed that alefacept had no negative impact on general quality of life, because the SF-36 did not show important changes. The authors then reached the conclusion that alefacept improved quality of life in patients with chronic plaque psoriasis and maintained this benefit for at least 12 weeks following cessation of treatment. This presentation of HRQL information is quite typical for trials of this sort (the trial was sponsored by the developer of alefacept). Unfortunately, most clinicians will have difficulty understanding what an improvement of 4.4 vs 1.8 or 3.4 vs 1.4 means to their patients. Does it mean that patients do feel substantially better or have noticed an important change, or does it mean that the improvement was small, not noticeable for patients, and statistically significant only because of the large sample size? The answer to this question is not clear because the authors do not provide satisfactory guidance as to how important these score changes are. However, the example should clarify one of the fundamental problems of HRQL data: the failure to present the data in interpretable and transparent manner. In the following paragraphs we will present possible solutions to these problems.

## Discussion

### Possible Solutions I – Dichotomized items

**Figure 1:**
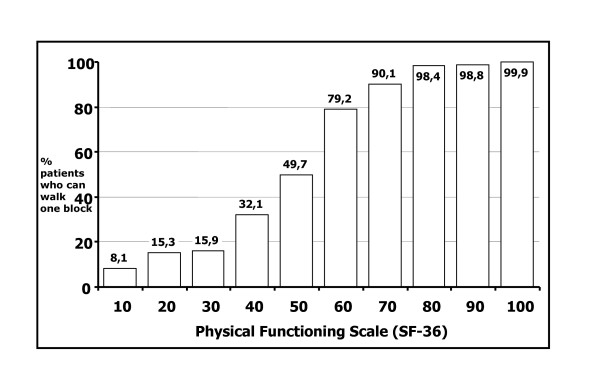
This figure shows how clinicians can use thresholds that either refer to an absolute score, (e.g. clinicians can consider all patients above a certain score as having achieved the outcome), or a change in score, (e.g. clinicians should consider patients' HRQL as having improved or deteriorated if they achieve a certain change in score on an instrument of interest). It shows the proportion of patients who are able, according to scores on the SF-36, to walk a distance of one block (approximately 100 meters) without difficulty. Increasing the score from 50 to 60 indicates that 29% more people state that they can walk without limitations. A score of 50 indicates that approximately 50% of patients are able to walk one block. An individual patient who has a score of 50 would have a 50% chance of being able to walk one block. If an intervention improved the score to 60, there would now be a 79% chance, or a 29% increase, of this patient's ability to walk one block.

Possible solutions to the problem of clinicians' lack of familiarity with HRQL scores exist. To focus on what is called the content-based interpretation of results proposed by Ware and Keller, let us take a look at the distribution of the SF-36 scores of the Physical Function scale based on the Medical Outcomes Study, a large study in the U.S. [9]. Figure 1 shows the proportion of patients who are able, according to scores on the SF-36, to walk a distance of one block (approximately 100 meters) without difficulty.

Figure 1 shows dichotomized responses to the item in a way that is meaningful. It also reveals differences across levels of the scale in the score range of interest. Increasing the score from 50 to 60 indicates that the proportion of respondents able to walk a block increased from 50% to 79%. Clinicians could, thus, interpret a score of 50 as a score that corresponds to approximately 50% of patients being able to walk one block. An individual patient who has a score of 50 would have a 50% chance of being able to walk one block. If an intervention improved the score to 60, there would now be a 79% chance, or a 29% increase, of this patient's ability to walk one block.

### Possible Solutions II – Item Response Theory

**Figure 2:**
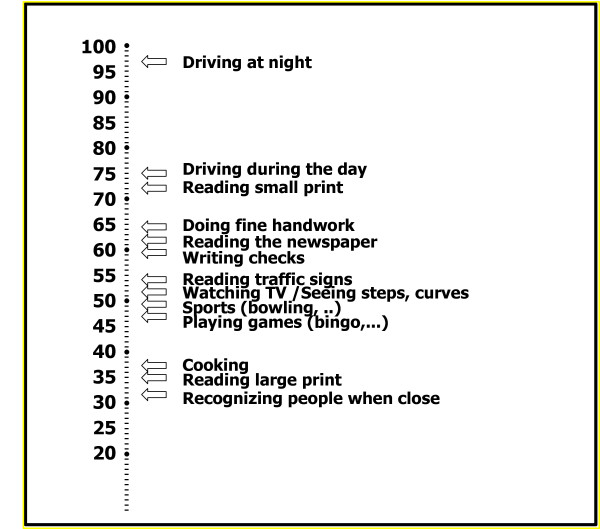
In this figure a score of 95 indicates that 50% of respondents can drive without difficulty during at night in regards to their visual function. A score of 32 indicates that 50% of respondents have no difficulty recognizing people when they are close. This interpretation provides clinicians and patients with guidance what they can expect based on a score on a multi-item instrument. The approach is restricted to instruments with a clear gradient of severity across the instruments' items.

Another example of the use of content-based interpretation of HRQL measures is the use of the visual function 14 index (VF14) [10]. This instrument asks respondents to rate the difficulties they have with their vision during performance of 14 everyday activities. Respondents answer on a five-points scale ranging from "no difficulty" to "unable to do the activity," and scores are then expressed on a 0 (worst function) to 100 (best function) scale. Valderas et al. used Rasch analysis based on item response theory (IRT) to estimate the item difficulty – they utilized a score on the item at which 50% of respondents can do the activity without difficulty [10]. Figure 2 indicates the scores on the VF14 that correspond to 50% of respondents being able to perform the described activity, ranked by importance or level of difficulty.

Figure 2 shows that a score of approximately 95 indicates that 50% of respondents can drive without difficulty at night in regard to their visual function. A score of 32 indicates that 50% of respondents have no difficulty recognizing people when they are close. The authors could have chosen a VF-14 score at which 25% of respondents have no difficulty recognizing people when they are close or a score at which 75% of respondents have no difficulty recognizing people when they are close. Using a cut-off of 50% simplifies interpretation because it implies a 1 to 1 chance.

Thus, the important contribution of IRT to the interpretation of HRQL scores is that it provides clinicians and patients with guidance what they can expect from respondents based on the entire score on a multi-item instrument. However, the IRT approach is restricted to instruments with a clear gradient of the level of difficulty across the instruments' items. It cannot be applied to situations when the response options there is no clear gradient in the severity of limitation or impairment associated with the items. The VF-14 works well in this regard because each item is more challenging than all lower items. Being able to drive at night, for instance, requires much better visual acuity than recognizing people even when they are close. The IRT approach to interpretability would not work for instruments in which this clear ordering does not exist.

### Possible solution III – use of minimal important difference

Different methods to determine the MID exist. Anchor-based methods rely on examining the associations between scores on the instrument that is under investigation and an anchor, an independent measure of HRQL that clinicians can easily interpret [11,12]. For instance, investigators have used global ratings of change for within-patient estimates of the MID. Another method is the use of between-patient ratings, that is, a comparison of one patient with another [13]. Research suggests that for three widely used HRQL instruments, the chronic respiratory questionnaire (CRQ), the asthma quality of life questionnaire (AQLQ) and the chronic heart failure questionnaire (CHQ), the MID is 0.5 on the 7 point scale [3-6].

However, two questions come to mind. First, does a group mean change of 0.6 in response to a treatment on the 7-point scale for the CRQ, AQLQ, or CHQ mean that all patients benefit? Second, does a group mean change of 0.3 mean that no patient benefits? In the following sections we will provide examples that will help answer these questions.

### Absolute score difference

Let us examine the Rankin stroke scale, an instrument widely used to measure dysfunction in patients who experienced a stroke [14]. The Rankin Stroke Scale has five levels: (1) no symptoms; (2) minor handicap – restriction in lifestyle, can look after self; (3) moderate handicap – restriction in lifestyle preventing independent existence; (4) moderately severe handicap – clearly preventing independence, no constant attention needed; and (5) severe handicap – requiring constant attention. A systematic review of RCTs examined the effects of thrombolysis on stroke using the Rankin Stroke Scale as an outcome [15]. The investigators used a threshold of a score of 2 (minor handicap) versus 3 (moderate handicap) and examined the proportion "dead or dependent" (dead or with a Rankin score of 3 or more). The results indicated that 55.2% in the thrombolysis and 68.3% in the control group experienced the outcome, representing a 42% odds reduction or 13.1% risk difference (absolute risk reduction). This risk difference translates into an NNT (1/risk difference) of 7 to 8 (or 130 fewer patients experiencing the event for every 1000 treated). That is, for 7 to 8 patients treated with thrombolysis in acute stroke, 1 fewer patient will be dead or dependent. This example shows how investigators can facilitate the interpretation of RCTs focusing on HRQL or functional outcomes by changing a categorical variable into a binary variable.

Another example is that of the use of neurolytic coeliac-plexus block (NCPB) versus systemic analgesic therapy (SAT) alone in unresectable pancreatic cancer [16]. The authors investigated the severity of pain using a 10-point scale. In the first six weeks, the number of patients reporting pain at an intensity of 5/10 or higher (moderate to severe pain), was significantly lower in the NCPB group (14% vs 40%) than in the SAT group. This result corresponded to a risk difference of 26%, an NNT (1/risk difference) of approximately 4, or 260 fewer patients for every 1000 treated who had pain that was moderate to severe. Here, the investigators have converted a continuous outcome into a binary outcome.

### Change Score Difference

**Figure 3:**
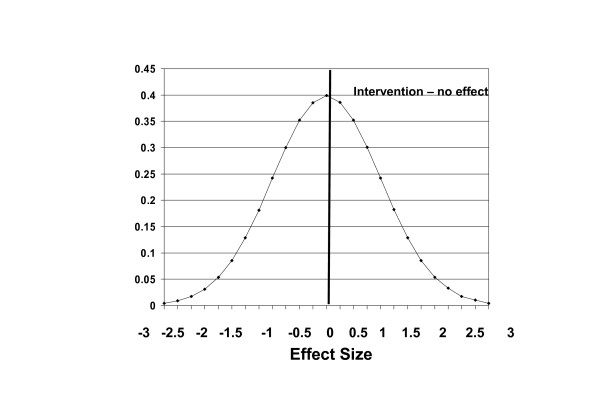
This figure shows the results of an intervention that, on average, has no effect and is expressed as effect size (mean difference divided by the standard deviation). It is evident that while the mean indicates no effect, the effect is normally distributed around the mean.

We will now examine examples of how clinicians can interpret change score differences. Figure 3 shows the results of an intervention that, on average, has no effect and is expressed as effect size (we will refer to effect size based on the standardized response mean calculated as difference divided by the standard deviation of the change score). It is evident that while the mean indicates no effect, the effect is normally distributed around the mean.

**Figure 4:**
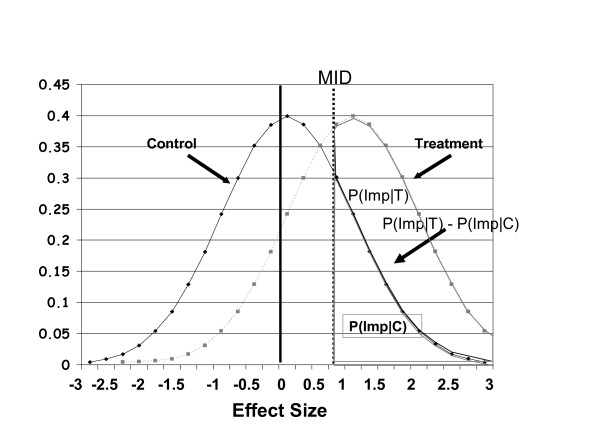
This figure shows the results of a hypothetical trial that compares the effects size after an effective intervention in a treatment group (T) to a control group (C) after an effective intervention. The treatment group had experienced a large effect. The control group had experienced, on average, a small improvement compatible with a placebo effect. The effects are normally distributed and not every patient improved; indeed, some deteriorated. The vertical line shows the hypothetical MID for patients participating in this trial. A certain proportion of patients in the control group show improvement greater than the MID [labeled P(Imp|C), the area under the curve to the right of the MID line in the control group]. In the treatment group, a larger proportion [P(Imp|T),] of patients show improvement greater than the MID (the area under the curve to the right of the MID line in the group receiving therapy). The difference between these two proportions [P(Imp|T) - P(Imp|C), the difference in the two areas under the curve] is the proportion of patients who improved above the MID after accounting for placebo or control group effects.

Figure 4 shows the results of a hypothetical trial that compares the effect size in a treatment group to a control group after an effective intervention. The treatment group experienced a large effect. The control group showed, on average, a small improvement compatible with a placebo effect. The effects are normally distributed and not every patient improved; indeed, some deteriorated.

The vertical line shows the hypothetical MID for patients participating in this trial. A certain proportion of patients in the control group [labeled P(Imp|C)] show improvement greater than the MID. In the treatment group, a larger proportion [P(Imp|T)] of patients show improvement greater than the MID. The difference between these two proportions [P(Imp|T) - P(Imp|C)] is the proportion of patients who improved above the MID after accounting for placebo or control group effects.

From this proportion one can easily calculate a risk differences or NNT. Norman et al showed a consistent relation between effect size and NNTs across a wide range of plausible specifications of the MID [17]. Table 1 presents the relation between NNT and effect size.

**Table 1:** This table shows the number needed to treat (NNT) by effect size

Effect Size	0.1	0.2	0.3	0.4	0.5	0.6	0.7	0.8
NNT	20	13	9	7	5	5	4	4

**Figure 5:**
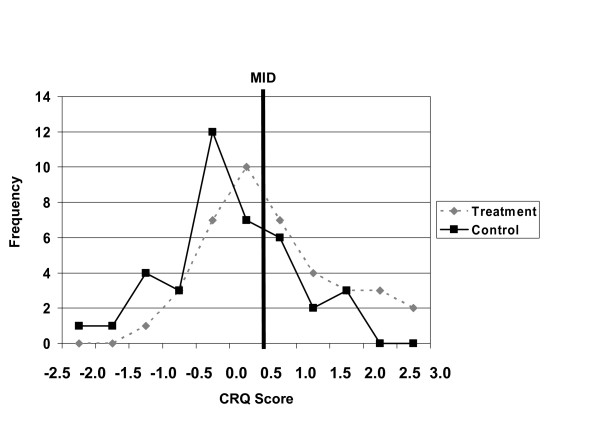
This example shows results from a randomized controlled trial comparing intensive respiratory rehabilitation to conventional care in patients with moderate to severe chronic respiratory disease. The lines depict change scores on the seven-point CRQ dyspnea domain. Because HRQL in patients with COPD deteriorates over time, the mean CRQ dyspnea score decreased (worse function) in the control group after three months of observation. Patients in the rehabilitation group showed a small increase in CRQ scores. The mean difference between the two groups was 0.6 (95% confidence interval = 0.18 to 1.03). The figure indicates that a greater proportion of patients in the treatment compared to the control group had an improvement greater than the MID. One can calculate the proportion of patients improving in both groups that improve above or below any threshold.

Figure 5 shows a real world example of how clinicians can interpret change score differences using the CRQ.

As we described above, the MID for the CRQ on each of the four HRQL domains is 0.5. This example shows results from a randomized controlled trial comparing intensive respiratory rehabilitation to conventional care in patients with moderate to severe chronic respiratory disease (COPD) [18]. The lines depict change scores on the seven-point CRQ dyspnea domain. Because HRQL in patients with COPD deteriorates over time, the mean CRQ dyspnea score decreased (worse function) in the control group after three months of observation. Patients in the rehabilitation group showed a small increase in CRQ scores. The mean difference between the two groups was 0.6 (95% confidence interval = 0.18 to 1.03). The figure indicates that a greater proportion of patients in the treatment compared to the control group had an improvement greater than the MID. One can calculate the proportion of patients improving in both groups that improve above or below any threshold.

## Conclusion

These examples of the interpretation of HRQL scores demonstrate that dichotomies may help the understanding of information provided by HRQL instruments in RCTs. There are several steps investigators can take to facilitate this process. They can choose thresholds and dichotomize responses on HRQL based on absolute scores or change scores to facilitate interpretation. Second, they can determine the MID of an HRQL instrument and provide mean differences and relate these to the MID. Finally, they can use the dichotomized responses and the MID to calculate the proportion of patients benefiting and transform these numbers into NNTs and natural frequencies. Simple dichotomies may bring HRQL information closer to the bedside.

## Competing interests

HJS and GG are authors of the CRQ. McMaster University and a research account used by HJS and GG receive licensing fees from the use of the CRQ.

## Authors' contributions

HJS prepared the first draft of this manuscript, and GG and EAA revised it critically and made suggestions for improvements. GG had the idea for the presentation on which this manuscript is based, HS made suggestions for improving the presentation.
